# Impact of thermal treatment on the quality, total antioxidant and antibacterial properties of fermented camel milk

**DOI:** 10.1038/s41598-025-91548-1

**Published:** 2025-03-12

**Authors:** Nagwa Hussein Ismail Abou-Soliman, Hagar Saeed Abd-Rabou, Sameh Awad, Amel A. Ibrahim

**Affiliations:** 1 Animal and Poultry Production Division, Department of Animal Breeding, Desert Research Centre, Cairo, Egypt; 2 Food Technology Department, Arid Lands Cultivation Research Institute, The City of Scientific Research and Technological Application (SRTA-City), Alexandria, Egypt; 3 Dairy Microorganisms and Cheese Research Laboratory (DMCR), Department of Dairy Science and Technology, Faculty of Agriculture, Alexandria University, Alexandria, Egypt

**Keywords:** Thermal treatments, Fermented camel milk, Viscosity, Fermentation, Antioxidant and antibacterial properties, Biochemistry, Biotechnology, Microbiology

## Abstract

This study investigated the impact of thermal treatments on the quality, antioxidant, and antibacterial properties of fermented camel milk during refrigerated storage. Two thermal treatment categories were applied: moderate (63 °C for 30 min and 72 °C for 15 s) and high (85 and 90 °C for 15 s and 30 min). Findings indicated that raw camel milk was found to be microbiologically unacceptable. Heating milk to 90 °C for 30 min notably reduced fermentation time, increased fermented milk viscosity, and altered whey protein electrophoretic patterns. While thermal treatments below 90 °C boosted lactic acid bacteria count in fermented milk compared to raw milk-derived samples (control), all treatments exceeded the recommended standards throughout storage. Thermal treatment decreased the proteolysis degree and DPPH radical scavenging activity but enhanced the ferric-reducing power of fermented milk compared to the control. Among, thermal treatments, samples from milk heated at 90 °C exhibited the highest scavenging activity and reducing power. Antibacterial efficacy against *Salmonella* Typhimurium surpassed that against *Escherichia coli* and *Staphylococcus aureus*; the most pronounced effect was observed in samples from milk heated at 90 °C. In conclusion, thermal treatment of camel milk is crucial to ensure sufficient hygiene for safe milk consumption. Heating camel milk at 90 °C before fermentation positively impacted most of the studied properties.

**Supplementary Information:**

The online version contains supplementary material available at 10.1038/s41598-025-91548-1.

## Introduction

According to the latest statistics of the Food and Agriculture Organization (FAO) for the year 2023, the global camel population is approximately 42.4 million, producing around 4.1 million tons of milk annually. Africa and the Middle East are the dominant producers, accounting for 74.8% of global camel milk production^1^. The global camel dairy product market has expanded significantly, with an estimated value of $14.1 billion in 2023, driven by increasing consumer awareness of the benefits of camel milk^2^.

Camel milk is recognized for its unique biological and therapeutic properties, including antioxidant and antimicrobial attributes, distinguishing it from other ruminants’ milk^3,4^. The antioxidant activity of camel milk is attributed to elevated vitamin C levels, surpassing that of cow milk by three to five times^5^. Additionally, camel milk contains various bioactive components, including caseins, whey proteins (particularly lactoferrin and α-lactalbumin), bioactive peptides, vitamins A and E, and minerals such as selenium and zinc. These components, along with antioxidant enzymes, collectively contribute to the overall antioxidant potential of camel milk^6^. The antimicrobial properties of camel milk are attributed to its higher content of antimicrobial factors, such as immunoglobulins, lactoferrin, lysozyme, lactoperoxidase, and peptidoglycan recognition protein, compared to cow milk^4,7^.

While some advocate for the health benefits of consuming raw camel milk, studies have raised concerns about its safety. Raw camel milk is frequently contaminated with coliform and pathogens such as *Staphylococcus aureus*, and *Salmonella* spp^8,9^. These issues are often related to inadequate hygiene practices during milk production, handling, and transportation^8^. Thermal treatment is commonly used in dairy industries to eliminate pathogens and spoilage-causing microorganisms, thereby ensuring the safety and microbial quality of milk and dairy products. Moreover, thermal treatment is the most commonly used processing tool to improve the body texture of fermented milk products via enhancing the denaturation of whey proteins and their interaction with casein micelles^10^.

The fermentation of camel milk poses significant challenges, including prolonged fermentation time and undesirable textural properties. Despite its importance, the impact of thermal treatment on the quality of camel dairy products, including fermented milk, remains limited^11^. In addition, there is a scarcity of research on the effects of thermal treatments on the antioxidant and antimicrobial activity of fermented camel milk. Previously, Abd Elhamid and Elbayoumi^12^ investigated the effect of applying a single temperature of 80 °C for extended periods of time up to 2 h (30, 60, 90, and 120 min) on the physicochemical, antioxidant, and antibacterial properties of camel milk yogurt. There is a lack of information on the effects of different temperatures and heating times on the antioxidant and antibacterial activity of fermented camel milk.

Therefore, the current study planned to explore the influence of moderate (63 °C for 30 min and 72 °C for 15 s) and high (85 and 90 °C for 15 s and 30 min) thermal treatments on the antioxidant and antibacterial properties of fermented camel milk, considering the microbial quality of raw and heated milk. The study also monitors the milk fermentation process, assesses the viscosity and microbial quality of the fermented milk, and tracks these properties over a 14-day storage period.

## Materials and methods

### Materials

Camel milk was obtained from Camel Research Center, Marsa Matrouh, Egypt. YOFLEX^®^ freeze-dried yogurt starter culture (YF-L904, 200U/1000 L), which consists of *Lactobacillus delbrueckii* subsp. *bulgaricus* and *Streptococcus thermophilus* were purchased from Chr. Hansen, Hoersholm, Denmark.

### Methods

#### Preparation of fermented milk

Fermented milk was prepared by the method of Tamime and Robinson^13^. Camel milk (total solids 11.0%, protein 3.2%, fat 3.4%, acidity 0.17%, and pH 6.5) was divided into seven portions. The first portion, without thermal treatment, was considered as a control. The other six portions were subjected to six thermal treatments: 63 °C for 30 min and 72 °C for 15 s (moderate thermal treatments) and 85 °C for 15 s and 30 min, and 90 °C for 15 s and 30 min (high thermal treatments). After thermal treatments, each portion was cooled to 42 °C and inoculated with 0.02% (w/v) of lyophilized yogurt starter culture, poured into plastic containers, and incubated at 42 °C until the pH of the samples reached 4.7 ± 0.05. During the fermentation period, the pH of the milk was monitored, and when the milk reached the desired pH, the fermentation time was recorded. Fermented milk samples were stored at 4 ± 1 °C and analyzed in triplicate after 1, 7, and 14 days of storage.

#### Chemical composition of milk

Milk was analyzed for total solids, protein, and fat (%) using the AOAC procedures^14^.

#### Titratable acidity and pH

The titratable acidity of milk and fermented milk was determined as lactic acid (%) according to AOAC^14^. The pH was determined by a digital pH meter (Martini, Italy).

#### Microbial quality

Microbial analysis of milk and fermented milk samples was performed according to ISO methods. Coliform was counted on violet-red bile lactose agar medium and incubated at 30 °C for 24 h^15^. *E. coli* was enumerated on Tryptone Bile X-glucuronide agar medium and incubated at 44 °C for 24 h^16^. Coagulase-positive *S. aureus* was counted on Baird-Parker agar medium and incubated at 37 °C for 48 h^17^. Yeasts and molds were enumerated on Dichloran Rose-Bengal Chloramphenicol agar medium and incubated at 25 °C for 5 days^18^. Cell viability of lactic acid bacteria (LAB) in fermented milk was determined by total plate count on De Man Rogosa Sharpe agar under micro-aerobic conditions for 48 h at 37 °C. Total plate count in milk was enumerated on plate count agar at 30 °C for 72 h^19^. Detection of *Salmonella* spp. in milk was performed according to ISO^20^.

#### Sodium Dodecyl sulfate-polyacrylamide gel electrophoresis

The changes in whey protein fractions due to thermal treatment were monitored in whey samples using sodium dodecyl sulfate-polyacrylamide gel electrophoresis (SDS-PAGE). SDS-PAGE, 12.5% T, was carried out under reducing conditions using a discontinuous buffer system described by Laemmli^21^. Whey samples from fermented milks at the end of fermentation were obtained by centrifugation at 10,000x*g* for 15 min at 4 °C. Whey samples were diluted (1:1) with reducing sample buffer, and 10 µL of each was applied to the gel. The molecular weight of protein bands was determined by Gel Analyzer 23.1 software.

#### Proteolysis degree

The degree of proteolysis in fermented milk supernatant was performed using the *O*-phthaldialdehyde (OPA) assay^22^. The supernatant was prepared according to the method of Shori and Baba^23^.

##### Supernatant Preparation

Ten grams of fermented milk were homogenized with 2.5 ml of distilled water and acidified to pH 4.0 using 0.1 M HCl. They were then held at 45 °C for 10 min and centrifuged at 10000xg for 10 min at 4 °C. The supernatant was adjusted to pH 7.0 using 0.1 M NaOH and re-centrifuged. It was kept at -20 °C until analysis. Before the assay, the supernatants were vortexed for 1 min and centrifuged at 4800x*g* for 5 min at 4 °C.

##### OPA assay

The OPA working reagent was made by blending 25 mL of 100 mM sodium tetraborate solution in distilled water, 2.5 mL of 20% (w/w) sodium dodecyl sulfate, 40 mg of OPA (dissolved in 1 mL of methanol) and 100 µL of β-mercaptoethanol. The volume was diluted to 50 mL with distilled water. Shortly, 150 µL of supernatant was added to 3.0 mL of OPA reagent in a quartz cuvette, then mixed briefly by inversion and incubated for precisely 2 min at room temperature. Absorbance was measured at 340 nm using a UV/visible spectrophotometer (UV/Visible Pg T80þ, England model) against blank (distilled water). Blank was done in the same manner as a sample. The degree of proteolysis was expressed as absorbance values.

#### Apparent viscosity

The apparent viscosity of fermented milk samples was determined using Rotary Viscometers (VR 3000 MYR viscometer, model V1). Viscosity was measured at 10 ± 1 °C using spindle No. 2 at a speed of 100 rpm.

#### Antioxidant activity

##### Preparation of water-soluble extract

The water-soluble extracts of fermented milk were prepared according to the method of Ayyash et al.^24^. Briefly, the pH of all fermented milk samples was adjusted to 4.6 using 1.0 M HCl or 1.0 M NaOH, followed by centrifugation at 10,000x*g* for 15 min at 4 °C. The supernatants were filtered through a 0.45-µm syringe filter (Polyethersulfone membrane) and stored at − 20 °C for further analyses. Before performing the assays, the water-soluble extracts were vortex-mixed for 1 min and centrifuged at 4800x*g* for 5 min at 4 °C.

##### Ferric reducing power

The ferric reducing power of the extracts was estimated by the method of Oyaizu^25^. A volume of 200 µL of extract was mixed with 500 µL of phosphate buffer (0.1 M, pH 6.6) and 500 µL of potassium ferricyanide (1% w/v). The mixture was incubated in a water bath at 50˚C for 20 min, then cooling to room temperature. Then, 500 µL of trichloroacetic acid (10% w/v) was added, followed by centrifugation at 4800x*g* for 10 min. Then, 500 µL of the supernatant was mixed with 500 µL of distilled water, and 100 µL of ferric chloride solution (0.1% w/v) was added. The mixture was incubated at room temperature for 30 min then the absorbance was measured at 700 nm using a UV/Visible spectrophotometer (UV/Visible Pg T80þ, England model) against blank (distilled water). Blank was done in the same manner as a sample. The reducing power was expressed as the absorbance values.

##### DPPH radical scavenging activity

The radical scavenging activity of extracts was determined with 1,1-diphenyl-2-picrylhydrazyl (DPPH) following the method described by Lim and Quah^26^. DPPH radical solution (0.004%, w/v) in 95% methanol was prepared. A volume of one mL of this solution was added to 500 µL of extract, mixed thoroughly, and incubated in the dark for 30 min at room temperature, then centrifuged at 4800x*g* for 5 min. Absorbance (Abs) of samples was measured at 517 nm against distilled water using a spectrophotometer (UV/Visible Pg T80þ, England model). Control was prepared by adding one mL of DPPH radical solution to 500 µL of methanol. The radical scavenging activity of the extracts was calculated as follows:

$${\text{Radical}}\;{\text{scavenging}}\;{\text{activity}}\;\% = \left( {1 - {\text{Abs}}_{{{\text{sample}}}} /{\text{Abs}}_{{{\text{control}}}} } \right) \times 100$$

#### Antibacterial activity

The standard well diffusion agar method was used to detect the antibacterial activity of fermented milk against *Salmonella enterica* subsp. *enterica* serovar Typhimurium ATCC14028, *S. aureus* ATCC6538, *E. coli* ATCC25922 and *Bacillus cereus* ATCC10876. All pathogens were incubated at 37 °C for 24 h, except for *B. cereus*, which was incubated at 30 °C for the same period. All strains were optimized at a concentration of 10^8^ CFU/mL. Next, 100 µL of each strain was inoculated into 100 mL of specific culture medium (Baird-Parker agar for *S. aureus*, violet red bile lactose agar for *E. coli* and *S.* Typhimurium, and Nutrient agar for *B. cereus*), and then poured in a Petri dish. The wells (9 mm in diameter) were performed. Fermented milk samples were added separately to the wells, and then the plates were incubated for 24 h at the optimum temperature of each pathogen. A volume of 100 µL of antibiotic [levofloxacin (250 mg/mL), Ampiflux (25 mg/mL), clindamycin (150 mg/mL), and tetracycline (250 mg/mL)] was used as a positive control for *S.* Typhimurium, *E. coli*, *S. aureus*, and *B. cereus*, respectively. The presence of an inhibition zone was considered as an antibacterial action. The diameter of the inhibition zone was measured in triplicate^27^.

#### Statistical analysis

Data were expressed as mean ± standard deviation. Data on microbial quality, acidity, and antibacterial activity were analyzed for statistical differences by a one-way analysis of variance (ANOVA). Other data were analyzed by two-way ANOVA (first factor: thermal treatment, second factor: storage time). Means were compared by Duncan’s test at a significance level of *P* < 0.05. Statistical analyses were performed using SAS, 2004 (SAS Institute, Inc., Cary, NC). Pearson’s correlation coefficient was used to determine the correlation.

## Results and discussion

### Milk

#### Fermentation process

**Fig. 1:**
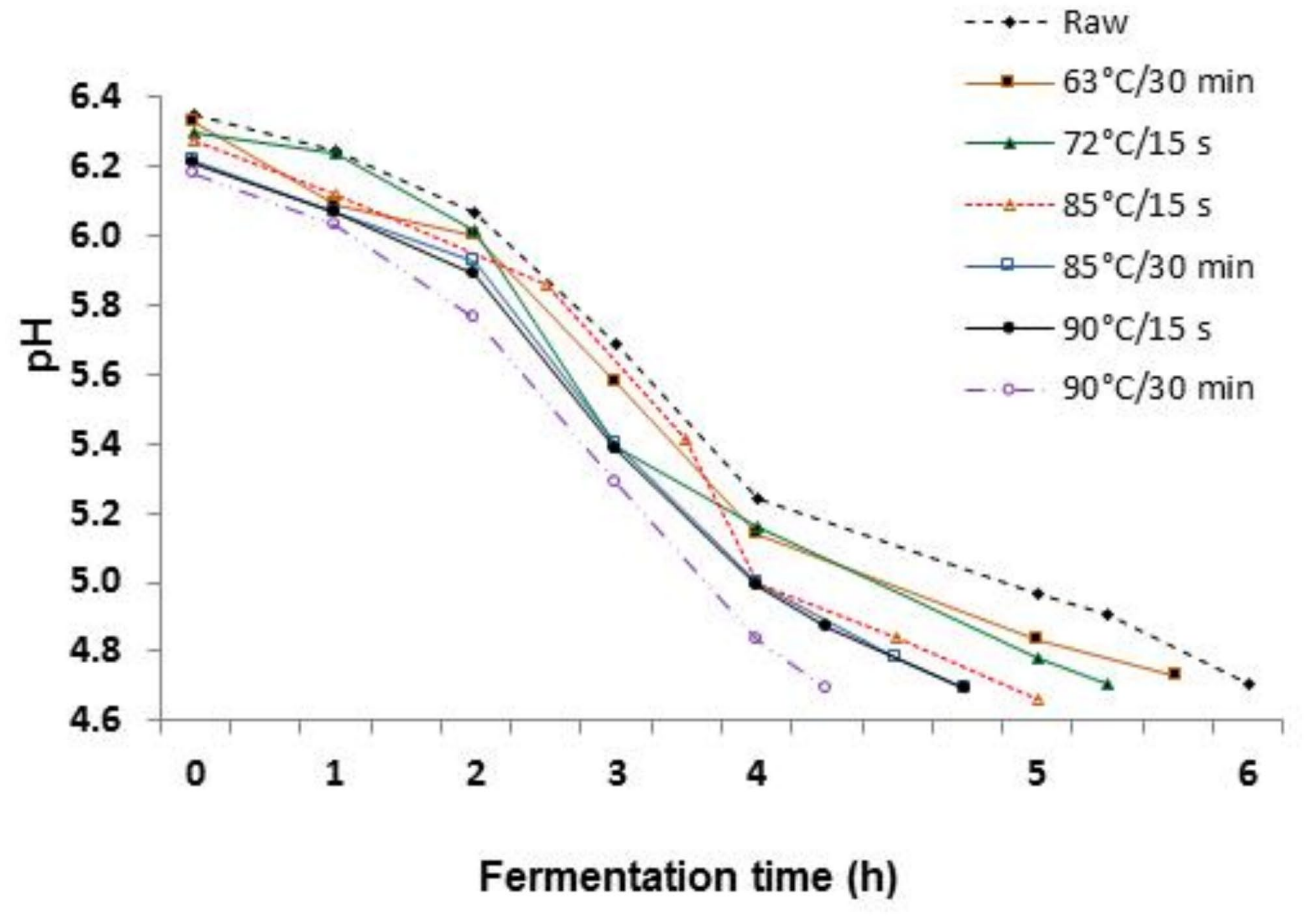
Fermentation time and acidification rate of camel milk subjected to different thermal treatments (63 °C for 30 min, 72 °C for 15 s, 85 and 90 °C for 15 s and 30 min).

The effect of thermal treatment of camel milk on the fermentation process (fermentation time and acidification rate) is illustrated in Fig. 1. It is evident that raw milk exhibited a longer fermentation time (6 h) and slower acidification rate compared to heat-treated milk samples. This phenomenon could be attributed to the elevated levels of immunoglobulins, lactoperoxidase, lactoferrin, and lysozyme in raw camel milk^7,28^, which may impede starter culture activity.

Figure 1 also shows that moderate thermal treatment (63 °C for 30 min or 72 °C for 15 s) slightly reduced the fermentation time of milk, likely due to the negligible effect of these treatments on the activity of antimicrobial proteins in milk^7^. Higher thermal treatments (85 °C and 90 °C for 15 s and 30 min) accelerated milk acidification rates and substantially reduced fermentation times. This could be attributed to the diminished biological activity of antimicrobial milk proteins, which further decreases with increasing heating temperature and time^7^. Notably, milk heated at 90 °C for 30 min showed the shortest fermentation time, lasting 4 h and 15 min. According to Bezie^29^, increased denaturation of whey proteins leads to decreased fermentation time. Additionally, lower starting pH values of high heat-treated milk may contribute to shorter fermentation time, as observed by Ozcan et al.^30^. A lower initial pH value of milk can reduce acid-base buffering (pH resistance), facilitating a pH decrease during fermentation^30^.

#### Microbial quality

The microbial quality of raw and heat-treated camel milk is presented in Table 1. Notably, raw milk exhibited a high plate count of 5.46 log CFU/mL, surpassing the value (3.92 log CFU/mL) reported by Ahmed et al.^31^ but falling short of the count (7.07 log CFU/mL) mentioned by Ombarak and Elbagory^32^. Furthermore, coagulase-positive *S. aureus* was detected at a significant level of 4.90 log CFU/mL. The presence of *S. aureus* in raw camel milk has also been previously reported by^8,9^, with mean counts of 2.74 and 2.54 log CFU/mL, respectively. Conversely, *Salmonella* spp. was not detected in raw milk.

**Table 1:** Microbial quality of raw and heat-treated camel milk.

Thermal treatment	Count (log CFU/mL)				
	Total viable count	Coliform	*E. coli*	Coagulase-positive *S. aureus*	Yeasts and molds
Raw	5.46 ± 0.15^a^	1.78 ± 0.08^a^	< 1	4.90 ± 0.05^a^	2.69 ± 0.09 ^a^
63 °C/30 min	2.90 ± 0.05^b^	< 1^b^	< 1	< 1^b^	< 1^b^
72 °C/15 s	2.00 ± 0.00^c^	< 1^b^	< 1	< 1^b^	< 1^b^
85 °C/15 s	1.99 ± 0.05^c^	< 1^b^	< 1	< 1^b^	< 1^b^
85 °C/30 min	1.51 ± 0.02^d^	< 1^b^	< 1	< 1^b^	< 1^b^
90 °C/15 s	1.62 ± 0.01^d^	< 1^b^	< 1	< 1^b^	< 1^b^
90 °C/30 min	1.16 ± 0.15^e^	< 1^b^	< 1	< 1^b^	< 1^b^

Data are presented as mean values ± standard deviation. Means with different small letters within a column are significantly different at *P* < 0.05.

Thermal treatments significantly reduced (*P* < 0.05) the total plate count in heated milk samples compared to raw milk (Table 1). Heating milk at 63 °C for 30 min resulted in a bacterial count of 2.90 log CFU/mL. However, heating milk at 90 °C for 30 min significantly decreased (*P* < 0.05) the bacterial count to 1.16 log CFU/mL. Coliform count also decreased from 1.78 log CFU/mL in raw milk to less than 1.0 log CFU/mL in all heated milk samples. This result confirms the efficiency of the thermal treatment. According to FAO Codex^33^, total bacteria and coliform counts in pasteurized camel milk should not exceed 5.0 log CFU /mL and 1 log CFU/ mL, respectively. The results of present study demonstrated that thermal treatments effectively reduced microbial counts to within recommended limits. Moreover, all thermal treatments had a similar impact on coagulase-positive *S. aureus*, yeast and molds counts, which were less than 1.0 log CFU/mL for all treatments, consistent with previous findings^34^.

### Fermented milk

#### The pH and titratable acidity

The pH and titratable acidity of fermented milk during storage are presented in Table 2. The results showed that fermented milk prepared from milk heated at 90 °C showed the lowest pH values compared to other treatments during cold storage. This finding is consistent with other studies, which demonstrated that severely heat-treated milk formulations developed higher acidities in yogurts^35^. Furthermore, Medeiros et al.^36^ hypothesized that protein denaturation promoted higher acidification rates in thermally fermented milk. Recently, Ayyash et al.^11^ observed that UHT-treated camel milk fermented with *Lb. plantarum* had the lowest pH, while fermented LTLT-treated camel milk had the highest pH during storage. The results also revealed a decrease in pH values and an increase in acidity values during storage due to continued acid production by the starter culture^11^.

**Table 2:** Effect of thermal treatment of camel milk on the pH, titratable acidity and microbial quality of fermented milk during 14 days of storage.

Thermal treatment	Storage time (day)	pH	Titratable acidity (%)	Count (log CFU/g)				
				Coliform	*E. coli*	Coagulase-positive *S. aureus*	Yeasts and Molds	Lactic acid bacteria
Raw	1	4.47	0.633 ± 0.01^aB^	4.26 ± 0.24^aA^	< 1	4.56 ± 0.24^aA^	3.26 ± 0.24^aA^	7.83 ± 0.04^bA^
	7	4.46	0.621 ± 0.01^eB^	3.26 ± 0.24^aB^	< 1	3.60 ± 0.00^aB^	2.95 ± 0.09^aA^	7.26 ± 0.24^cB^
	14	4.45	0.651 ± 0.01^dA^	2.74 ± 0.24^aC^	< 1	< 1	3.36 ± 0.32^aA^	7.08 ± 0.19^bB^
63 °C/30 min	1	4.62	0.579 ± 0.01^cC^	< 1	< 1	< 1	< 1	7.69 ± 0.09^cB^
	7	4.58	0.612 ± 0.00^fB^	< 1	< 1	< 1	< 1	8.56 ± 0.24^aA^
	14	4.44	0.651 ± 0.01^dA^	< 1	< 1	< 1	< 1	8.48 ± 0.44^aA^
72 °C/15 s	1	4.43	0.633 ± 0.00^aB^	< 1	< 1	< 1	< 1	7.90 ± 0.05^abB^
	7	4.42	0.645 ± 0.01^cAB^	< 1	< 1	< 1	< 1	8.46 ± 0.15^abA^
	14	4.42	0.657 ± 0.01^dA^	< 1	< 1	< 1	< 1	8.39 ± 0.36^aA^
85 °C/15 s	1	4.40	0.609 ± 0.01^bC^	< 1	< 1	< 1	< 1	7.98 ± 0.03^aA^
	7	4.40	0.630 ± 0.00^dB^	< 1	< 1	< 1	< 1	8.39 ± 0.36^abA^
	14	4.39	0.672 ± 0.01^cA^	< 1	< 1	< 1	< 1	8.39 ± 0.36^aA^
85 °C/30 min	1	4.52	0.576 ± 0.01^cB^	< 1	< 1	< 1	< 1	7.93 ± 0.08^aA^
	7	4.52	0.585 ± 0.00^gB^	< 1	< 1	< 1	< 1	8.08 ± 0.19^bA^
	14	4.41	0.681 ± 0.01^cA^	< 1	< 1	< 1	< 1	8.00 ± 0.00^aA^
90 °C/15 s	1	4.48	0.572 ± 0.01^cB^	< 1	< 1	< 1	< 1	7.95 ± 0.05^aA^
	7	4.24	0.720 ± 0.00^aA^	< 1	< 1	< 1	< 1	7.08 ± 0.19^cB^
	14	4.24	0.714 ± 0.01^bA^	< 1	< 1	< 1	< 1	6.98 ± 0.03^bB^
90 °C/30 min	1	4.35	0.609 ± 0.01^bC^	< 1	< 1	< 1	< 1	6.98 ± 0.03^dB^
	7	4.22	0.684 ± 0.00^bB^	< 1	< 1	< 1	< 1	7.26 ± 0.24^cAB^
	14	4.22	0.732 ± 0.01^aA^	< 1	< 1	< 1	< 1	7.46 ± 0.15^bA^
Guideline				≤ 1^*^	Free	Free /10 g	NA	≥ 6

Data are presented as mean values ± standard deviation. Means with different small letters at the same day of storage in the column are significantly different at *P* < 0.05, means with different capital letters within the same treatment in the column are significantly different at *P* < 0.05. * Codex 243:2010^37^; Egyptian standards 8042:2016^38^; NA: Not applicable.

#### SDS-PAGE

**Fig. 2:**
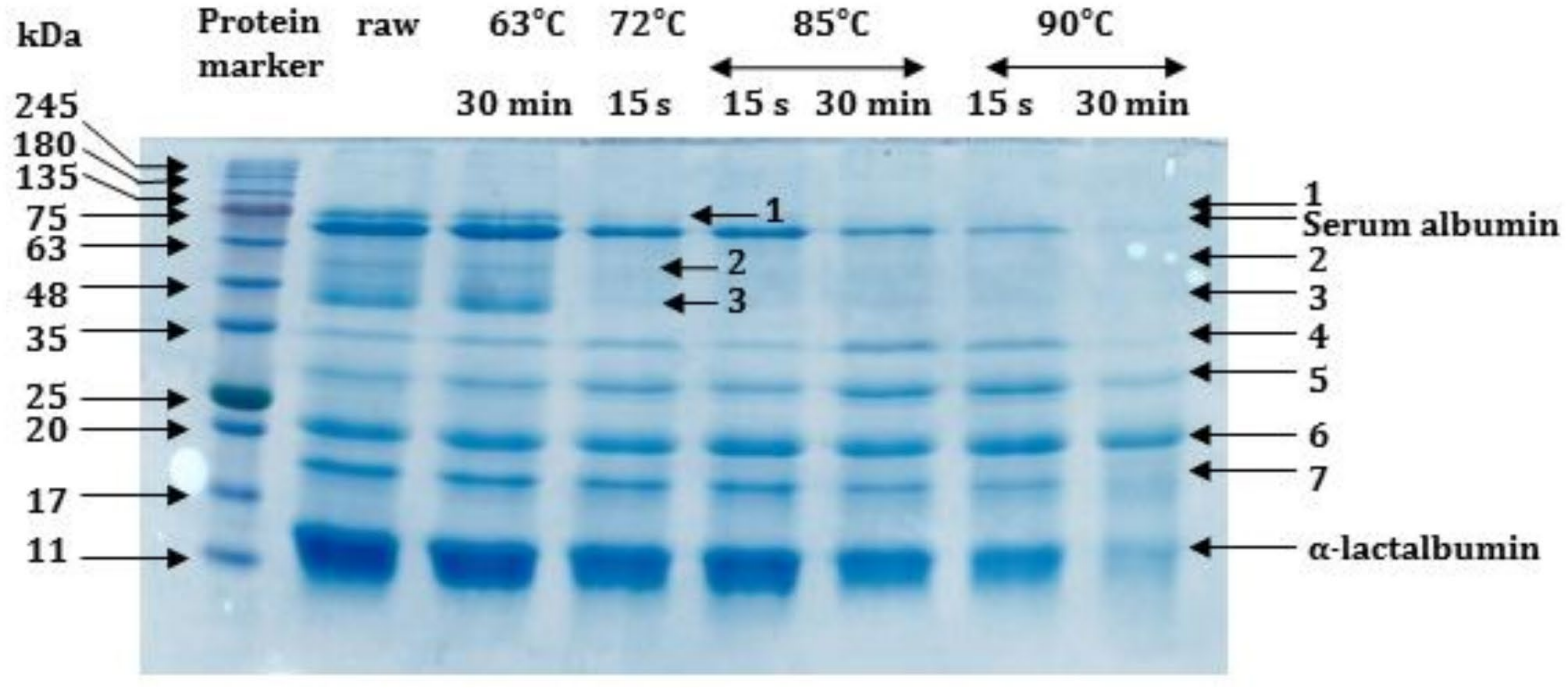
SDS-PAGE electrophoretic patterns of whey samples prepared from fermented camel milks at the end of fermentation. Fermented milk samples were made from raw milk and milk heated at 63 °C/30 min, 72 °C/15 s, 85 °C/15 s, 85 °C/30 min, 90 °C/15 s, and 90 °C/30 min.

The SDS-PAGE electrophoretic patterns of fermented milk whey at the end of fermentation are illustrated in Fig. 2. The electrophoretic pattern of the raw sample showed that α-lactalbumin was the dominant component of whey proteins followed by serum albumin^39^. The estimated molecular weights (MW) of α-lactalbumin and serum albumin were 12.0 and 66.5 kDa, respectively^40,41^. The electrophoretic pattern of the raw sample also shows the presence of other protein bands (labeled on the resolving gel as 1, 2, 3, 4, 5, 6, and 7) separated on the gel with MW of 75.0, 54.0, 43.0, 34.5, 27.0, 19.5, and 17.0 kDa, respectively.

SDS-PAGE results showed a diverse effect of thermal treatments on whey proteins. In general, increasing thermal treatment intensity led to a decrease in protein bands intensity except for band 6, which was the most stable protein during all thermal treatments. On the contrary, bands 2 and 3 were the most heat-sensitive proteins, disappearing after heating milk at 72 °C for 15 s.

Figure 2 shows that heating milk at 63 °C for 30 min before fermentation had no significant effect on whey protein electrophoretic patterns, aligning with previous findings^7^. Heating the milk at 72 °C for 15 s or 85 °C for 15 s altered the protein patterns to some extent, with a decrease in serum albumin and α-lactalbumin. However, heating milk at 85 °C for 30 min resulted in a further decrease in serum albumin and α-lactalbumin and an increase in the intensity of bands 4 and 5. Heating milk to 90 °C, particularly for 30 min, resulted in drastic changes in protein electrophoretic patterns; some proteins almost disappeared (serum albumin, band 4, and band 7), and others appeared as very faint bands (band 5 and α-lactalbumin). These changes indicate protein interactions and polymer formation due to heating. In cows and other ruminants, heating milk leads to the denaturation of whey proteins and the formation of whey protein-casein complexes, which increase with increasing heating temperature and time^42^. In these milks, β-lactoglobulin is primarily responsible for these interactions. However, the mechanism of these interactions in camel milk is unknown due to its lack of β-lactoglobulin.

#### Proteolysis degree

**Fig. 3:**
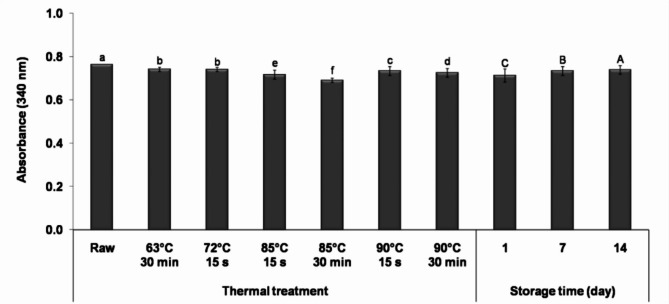
Proteolysis degree during 14 days of cold storage in fermented camel milk prepared from milk subjected to different thermal treatments (63 °C for 30 min, 72 °C for 15 s, 85 and 90 °C for 15 s and 30 min). Data are presented as mean values ± standard deviation. Means with different small letters are significantly different at *P* < 0.05; mean values with different capital letters are significantly different at *P* < 0.05.

The impact of thermal treatment on the proteolysis degree in fermented milk is depicted in Fig. 3. Notably, raw milk-derived samples exhibited a significantly higher proteolysis degree (*P* < 0.05) than those prepared from heat-treated milk. This could be attributed to the elevated microbial load and naturally occurring enzymes in raw milk.

Thermal treatment affected the degree of proteolysis, with moderate treatments yielding higher proteolysis than high thermal treatments. No significant difference (*P* > 0.05) was observed between samples from milk heated at 63 °C for 30 min or 72 °C for 15 s. However, increased heating time at 85 and 90 °C significantly (*P* < 0.05) reduced proteolysis. This aligns with previous findings indicating a decline in proteolytic activities of yogurt cultures with prolonged time-temperature heating conditions^43^. Fermented milk from moderate heat-treated milk exhibited higher proteolysis, likely due to the unfolding of whey proteins, facilitating enzymes’ access to cleavage sites. Conversely, high thermal treatment resulted in lower proteolysis, possibly due to the formation of casein-whey protein complexes, hindering enzymes from attacking cleavage sites buried within protein aggregates^44^. On the other hand, heating milk at 90 °C before fermentation significantly (*P* < 0.05) increased proteolysis compared to 85 °C, likely due to structural changes making proteins more susceptible to proteolysis. Previous research supported this, demonstrating loose and reticular structures at higher temperatures^45^.

During cold storage, a significant increase (*P* < 0.05) in the degree of proteolysis was observed. Amino acid Liberation by *Lactobacillus delbrueckii* subsp. *bulgaricus* is crucial for the growth of *Streptococcus thermophilus*^46^.

#### Apparent viscosity

**Fig. 4:**
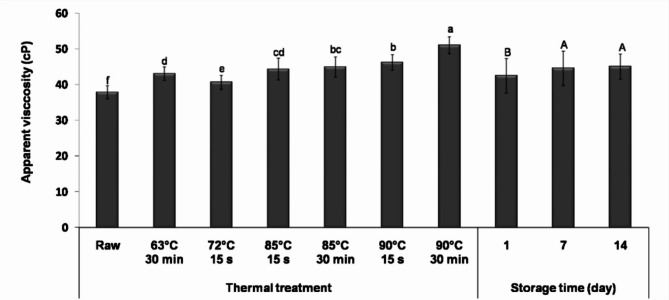
Apparent viscosity during 14 days of cold storage in fermented camel milk prepared from milk subjected to different thermal treatments (63 °C for 30 min, 72 °C for 15 s, 85 and 90 °C for 15 s and 30 min). Data are presented as mean values ± standard deviation. Means with different small letters are significantly different at *P* < 0.05; mean values with different capital letters are significantly different at *P* < 0.05.

The apparent viscosity of fermented camel milk during storage is shown in Fig. 4. Thermal treatment significantly improved the viscosity of fermented milk, which increased with the intensity of thermal treatment (*P* < 0.05). Samples from milk heated at 90 °C for 30 min exhibited the highest viscosity throughout storage. It is noteworthy that although thermal treatment improved viscosity, moderate heat-treated milk samples retained a watery consistency. This is primarily attributed to the absence of β-lactoglobulin and low κ-casein concentration (3.47% of total casein) in camel milk^47^.

The increased apparent viscosity with thermal treatment intensity could be explained by the increased denaturation of whey proteins and their interactions with caseins, reinforcing the acid-induced gel network structure. These reactions were more pronounced with increased thermal treatment intensity, as evidenced by electrophoresis results (Fig. 2) and corroborated by Ayyash et al.^11^. The differences in viscosity among treatments may also be attributed to differences in the acidity values of fermented milk samples. Pearson’s correlation analysis revealed a moderate correlation between viscosity and acidity values of fermented milk *(r* = 0.395, *P* = 0.0014). This finding aligns with Lucey and Singh^10^, who reported that acidity and low pH resulting from the LAB fermentation led to structural changes responsible for acid-induced milk gels’ rheological properties. After 14 days of storage, a significant increase (*P* < 0.05) in viscosity was observed in all samples, possibly due to continued acid production and protein aggregation.

#### Antioxidant activity

##### Ferric reducing power

**Fig. 5:**
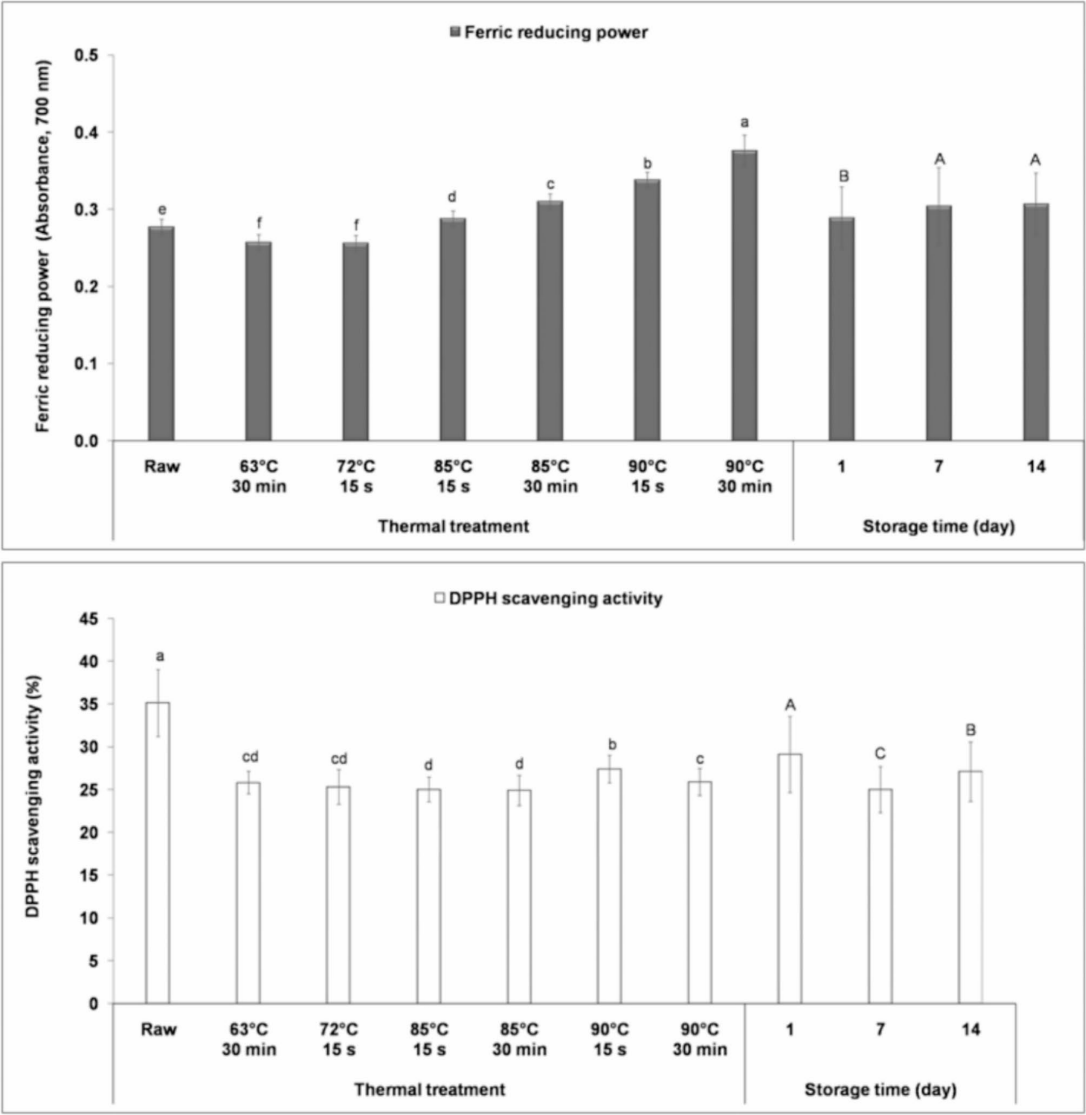
Antioxidant activity during 14 days of cold storage in fermented camel milk prepared from milk subjected to different thermal treatments (63 °C for 30 min, 72 °C for 15 s, 85 and 90 °C for 15 s and 30 min). Data are presented as mean values ± standard deviation. Means with different small letters are significantly different at *P* < 0.05; mean values with different capital letters are significantly different at *P* < 0.05.

The impact of thermal treatment on the ferric-reducing power of fermented milk is illustrated in Fig. 5. The results showed that moderate thermal treatments resulted in a significant decrease (*P* < 0.05) in the reducing power of fermented milk compared to samples prepared from raw milk, consistent with previous studies on milk^48^. This decrease may be attributed to the degradation of some naturally occurring reductants, such as phenolic compounds and ascorbic acid. Alyaqoubi et al.^48^ reported that pasteurized milk had significantly lower total phenolic content than raw milk.

However, high thermal treatments (85 and 90 °C) resulted in a significant increase (*P* < 0.05) in the reducing power of fermented milk compared to control samples and those prepared from moderate heat-treated milk, with a greater increase observed with prolonged heating time. The highest reducing power value was recorded with samples prepared from milk heated at 90 °C for 30 min. This finding is supported by previous studies, which reported that heating milk at 90 °C for 10 min resulted in higher ferric-reducing antioxidant power than raw milk^49^. The increase in reducing power may be due to the formation of new reductants, such as Maillard reaction products, which have antioxidant properties due to their reductone structures with strong reducing power and metal complexing properties^50^. Notably, no correlation was found between the reducing power and the degree of proteolysis (*P* > 0.05).

The results also showed that the reducing power of all fermented milk samples increased significantly (*P* < 0.05) at the end of storage. This finding is consistent with previous studies, which reported an increase in the reducing power of yogurt during storage^51^ due to the formation of reductants by the starter culture such as organic acids^52^.

##### DPPH radical scavenging activity

Figure 5 illustrates the adverse effect of thermal treatment on the DPPH radical scavenging activity of fermented milk. Samples prepared from raw milk had the highest scavenging activity (*P* < 0.05) throughout the storage period. This is due to the high levels of vitamin C, lactoferrin, α-lactalbumin, and immunoglobulins in camel milk, which can neutralize and scavenge free radicals^53^. Thermal treatment decreased scavenging activity, likely due to thermal degradation of water-soluble vitamins and proteins responsible for radical scavenging activity^29^. Previous studies on milk reported that pasteurization or heating milk at 90 °C for 10 min decreased DPPH radical scavenging^48,49^.

On the other hand, samples derived from milk heated at 90 °C showed the highest radical scavenging activity, which decreased with increasing heating time. In contrast, samples from milk heated at 85 °C for 15 s or 30 min had the lowest radical scavenging activity. The differences in scavenging activity among samples may be partly attributed to peptides released due to proteolysis. Pearson’s correlation analysis revealed a moderate correlation between the degree of proteolysis and DPPH radical scavenging activity (*r* = 0.395, *P* = 0.001). The results also indicated that storage significantly impacted the free radical scavenging activity, with the highest values recorded on the first day, consistent with the findings of Li et al.^54^.

In conclusion, this study demonstrates that thermal treatments decreased the free radical scavenging activity of fermented camel milk. Notably, samples made from raw milk exhibited the highest free radical scavenging activity during storage. However, microbiological analysis revealed that raw camel milk poses safety risks, underscoring the necessity of thermal treatment prior to fermentation. High-temperature treatments significantly enhanced the ferric-reducing power of fermented milk compared to both raw fermented milk and those prepared from moderately heat-treated milk. Among thermal treatments, fermented milk derived from camel milk heated to 90 °C showed the highest antioxidant activity, as measured by free radical scavenging activity and ferric reducing power. This increased antioxidant potential may confer health benefits to consumers, as a diet rich in antioxidants has been linked to a reduced risk of chronic diseases, including cancer, diabetes, and cardiovascular conditions^55^.

#### Antibacterial activity

The antibacterial activity of fermented camel milk during storage is presented in Table 3. The agar well diffusion method was used to evaluate the antibacterial activity of fermented camel milk against *B. cereus*, *E. coli*, *S. aureus* and *S*. Typhimurium. The results indicated that fermented milk exhibited no antibacterial effect against *B. cereus*. In contrast, it displayed inhibition zone against *E. coli*, *S*. Typhimurium, and *S. aureus*. Notably, the antibacterial effect of fermented milk against *S.* Typhimurium was significantly higher (*P* < 0.05) than its effect against the other two pathogens. These findings are consistent with previous studies on buffalo milk yogurt^56^ Camel milk has been shown to possess bactericidal and bacteriostatic effects against both Gram-negative and Gram-positive bacteria^28^. Additionally, fermented camel milk has demonstrated antimicrobial effects against various pathogens^57^.

Table 3 summarizes the impact of the thermal treatment on the antibacterial activity of fermented camel milk against *E. coli*, *S*. Typhimurium, and *S. aureus*. Thermal treatments had no significant impact (*P* > 0.05) on the antibacterial activity of fermented milk against *E. coli* and *S. aureus* compared to the control with inhibition zone of 20 and 10 mm, respectively. However, the inhibitory effect of fermented milk against *S.* Typhimurium increased significantly (*P* < 0.05) with the intensity of thermal treatment. Fermented milk from milk heated at 90 °C exhibited the highest antibacterial activity against *S.* Typhimurium. Similar results have been reported by other studies, which found that thermal treatment can enhance the antibacterial activity of fermented goat milk^58^.

**Table 3:** Effect of thermal treatment of camel milk on the antibacterial activity of fermented milk during 14 days of refrigerated storage.

Thermal treatment	Diameter of inhibition zone (mm)								
	*E. coli*			*S.* Typhimurium			*S. aureus*		
	Storage time (day)			Storage time (day)			Storage time (day)		
	1	7	14	1	7	14	1	7	14
Raw	20.0 ± 0.00^bA^	20.0 ± 0.00^bA^	20.0 ± 0.00^bA^	18.5 ± 0.50^cB^	20.0 ± 0.00^cA^	20.0 ± 0.00^cA^	10.0 ± 0.00^aA^	10.0 ± 0.00^aA^	10.0 ± 0.00^aA^
63 °C/ 30 min	20.0 ± 0.00^bA^	20.0 ± 0.00^bA^	20.0 ± 0.00^bA^	21.0 ± 0.00^bC^	22.0 ± 0.00^bB^	23.0 ± 0.00^bA^	10.0 ± 0.00^aA^	10.0 ± 0.00^aA^	10.0 ± 0.00^aA^
72 °C/ 15 s	22.0 ± 0.00^aA^	22.0 ± 0.00^aA^	21.7 ± 0.58^aA^	22.0 ± 0.00^aB^	22.0 ± 0.00^bB^	23.0 ± 0.00^bA^	10.0 ± 0.00^aA^	10.0 ± 0.00^aA^	10.0 ± 0.00^aA^
85 °C/ 15 s	20.0 ± 0.00^bA^	20.0 ± 0.00^bA^	20.0 ± 0.00^bA^	22.0 ± 0.00^aB^	22.0 ± 0.00^bB^	23.0 ± 0.00^bA^	10.0 ± 0.00^aA^	10.0 ± 0.00^aA^	10.0 ± 0.00^aA^
85 °C/ 30 min	20.0 ± 0.00^bA^	20.0 ± 0.00^bA^	20.0 ± 0.00^bA^	22.0 ± 0.00^aB^	22.0 ± 0.00^bB^	23.0 ± 0.00^bA^	10.0 ± 0.00^aA^	10.0 ± 0.00^aA^	10.0 ± 0.00^aA^
90 °C/ 15 s	20.0 ± 0.00^bA^	20.0 ± 0.00^bA^	20.0 ± 0.00^bA^	22.7 ± 0.58^aB^	23.0 ± 0.00^aBA^	23.7 ± 0.58^aA^	10.0 ± 0.00^aA^	10.0 ± 0.00^aA^	10.0 ± 0.00^aA^
90 °C/ 30 min	20.0 ± 0.00^bA^	20.0 ± 0.00^bA^	20.0 ± 0.00^bA^	22.7 ± 0.58^aB^	23.0 ± 0.00^aBA^	23.7 ± 0.58^aA^	10.0 ± 0.00^aA^	10.0 ± 0.00^aA^	10.0 ± 0.00^aA^

Data are presented as mean values ± standard deviation. Means with different small letters within a column are significantly different at *P* < 0.05; mean values with different capital letters within a row are significantly different at *P* < 0.05.

The antimicrobial activity of raw fermented milk is primarily attributed to its high concentration of antimicrobial substances, including immunoglobulins, lactoferrin, and lysozyme^7^. Therefore, it was expected that the antibacterial activity of fermented milk might decrease with the intensity of thermal treatment. However, the results showed that thermal treatment had no negative effect on the antibacterial activity of fermented milk against *E. coli* and *S. aureus*. Moreover, the antibacterial activity against *S.* Typhimurium increased with higher thermal treatment. This result may be attributed to the acidity of fermented milk, which outweighed the negative effect of thermal treatment on the antimicrobial substances in camel milk. Pearson’s correlation analysis revealed a significant correlation between the antibacterial activity against *S.* Typhimurium and the acidity of fermented milk (*r* = 0.442, *P* = 0.000). This result aligns with the earlier findings of Nassib et al.^59^, which demonstrated that acidity was a critical factor in suppressing *S*. Typhimurium growth in buffalo milk yogurt. Conversely, no correlation was observed between acidity and antibacterial activity of fermented milk against *E. coli* and *S. aureus.* Furthermore, no significant relationship (*P* > 0.05) was found between proteolysis and antibacterial activity in fermented milk.

The results also showed that storage significantly enhanced the antibacterial activity of fermented camel milk against *S*. Typhimurium (*P* < 0.05), consistent with previous findings in buffalo milk yogurt^59^. In contrast, storage had no significant effect on antibacterial activity against *E. coli* and *S. aureus* (*P* > 0.05).

#### Microbial quality

The microbial quality of fermented milk during storage is presented in Table 2. Fermented raw milk contained coliform, coagulase-positive *S. aureus*, yeasts, and molds with counts of 3.42, 4.08, and 3.19 log CFU/g, respectively, which exceeded the microbial standard recommended by Codex^37^, and Egyptian standards^38^ for fermented cow milk. Thermal treatment significantly (*P* < 0.05) improved the microbial quality of fermented milk. All fermented milk prepared from heat-treated milk had a count of coliform, coagulase-positive S. aureus, yeasts, and molds of less than 1.0 log CFU/g. During cold storage, the LAB count was significantly (*P* < 0.05) increased in fermented milk from milk heated at 63 °C for 30 min up to 85 °C for 15 s compared to control and other thermal treatments. In these samples, the count of LAB increased on day 7 and remained stable until the end of storage. Heating milk at 90 °C decreased the LAB count of fermented milk compared to other treatments, possibly due to the development of acidity. The LAB count of all fermented milk exceeded the recommended standard of 6.0 log CFU/g during storage^37,38^. Studies on the effect of thermal treatments on the count of LAB are very few and conflicting. Studies showed that bacterial growth in milk was inhibited or progressively weaker with increasing heating intensity^60,61^. However, other studies demonstrated improved LAB growth in heated milk with increasing heating intensity^62^.

## Conclusions

This study highlights the risks of consuming raw camel milk and its fermented products, emphasizing the need of thermal treatment to ensure safety. Thermal treatment, particularly at 90 °C for 30 min, improved the quality of fermented milk by reducing fermentation time, a crucial factor in fermented milk production, and increasing viscosity. Additionally, thermal treatments, even those below 90 °C, positively affected the microbial quality of fermented milk, particularly by enhancing the count of lactic acid bacteria compared to the untreated samples. While thermal treatment decreased free radical scavenging activity, high-thermal treatments increased ferric-reducing power. Among thermal treatments, fermented milk derived from camel milk heated to 90 °C showed the highest free radical scavenging activity and ferric reducing power. This increased antioxidant potential may confer health benefits to consumers. Further, heating milk at 90 °C enhanced the antibacterial potential against *S*. Typhimurium. Overall, heating camel milk at 90 °C for 30 min emerged as the most suitable thermal treatment for fermented camel dairy industries for optimizing the studied qualities. However, further research is needed to determine the effect of this treatment on the biological value of the fermented product.

## Electronic supplementary material

Below is the link to the electronic supplementary material.

Supplementary Material 1

## Data Availability

All data generated or analyzed during this study are included in this published article.
