# Supplementary material for: Impact of thermal treatment on the quality, total antioxidant and antibacterial properties of fermented camel milk
Source: Sci Rep. 2025 Mar 12;15:8533. doi: 10.1038/s41598-025-91548-1 (PMC11903780; doi:10.1038/s41598-025-91548-1)
Supplement: Supplementary file 1 — Supplementary Material 1 [file 41598_2025_91548_MOESM1_ESM.pdf]

Supplementary File

Raw gel electrophoresis Chromatogram .

Different shots were taken for the same chromatogram.

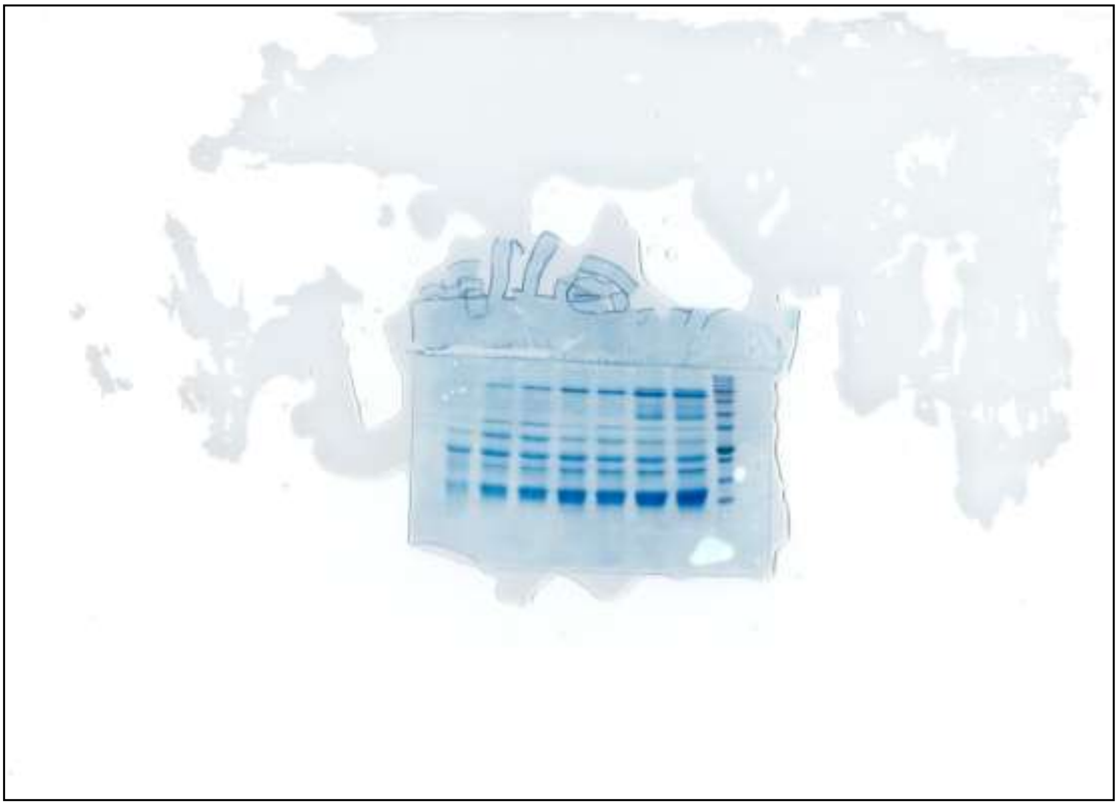

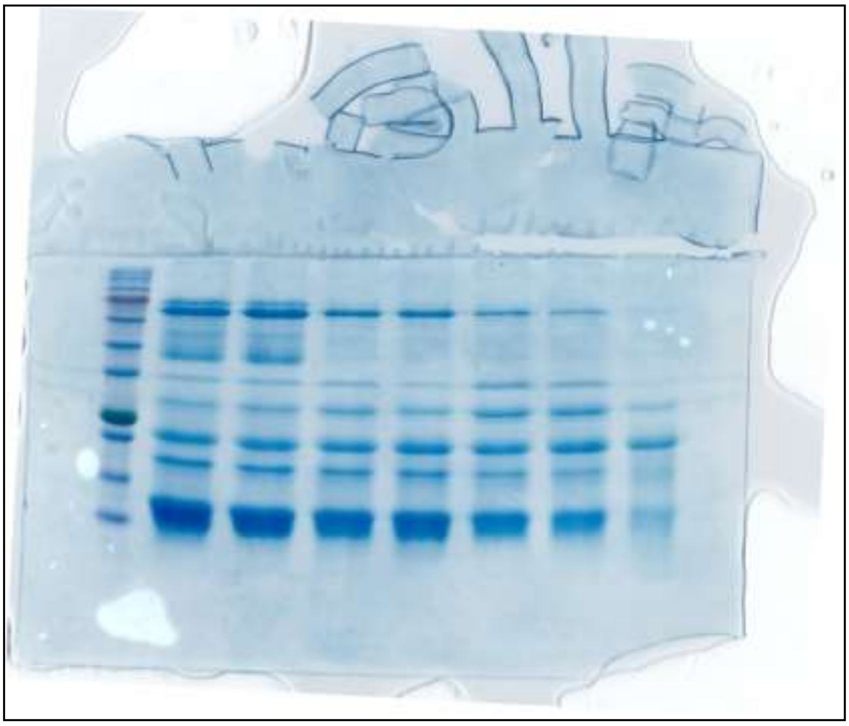

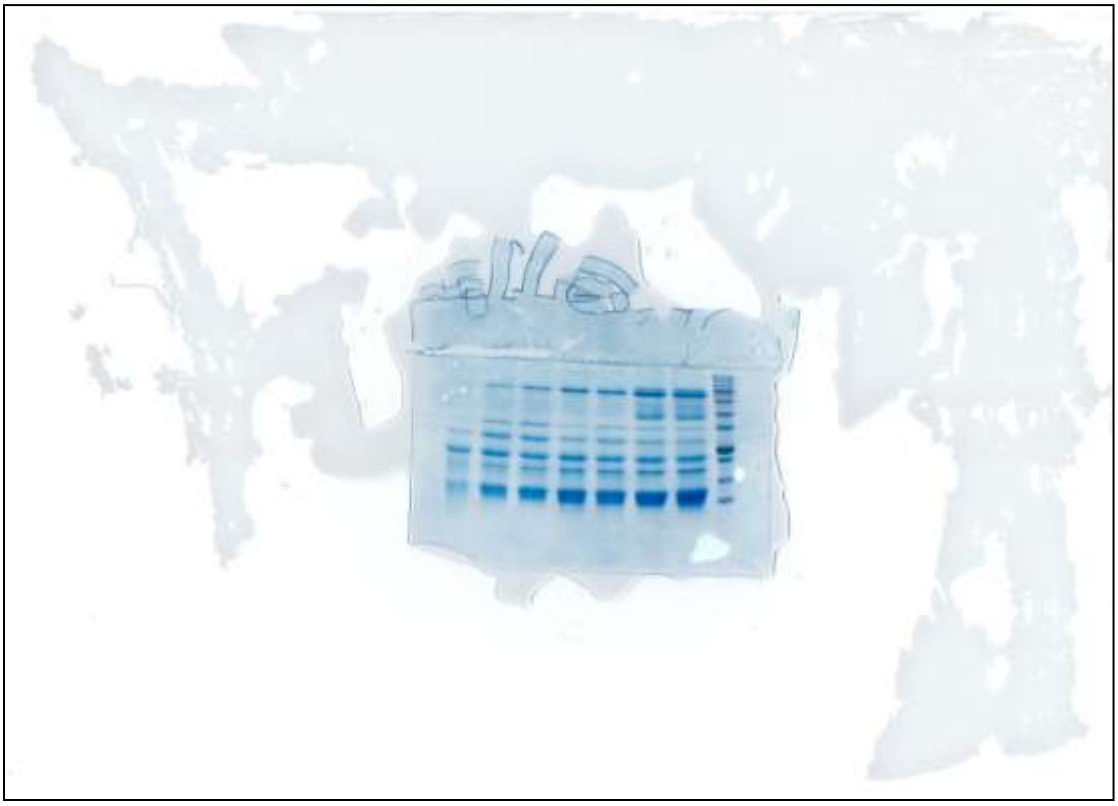

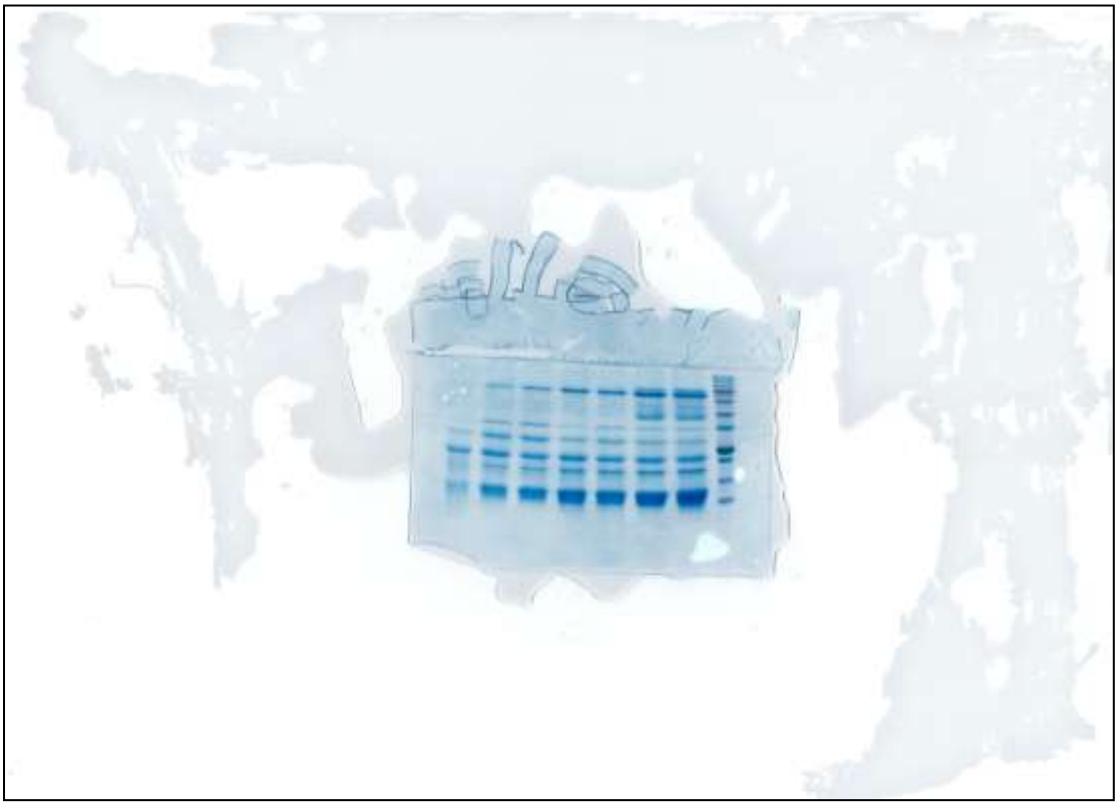

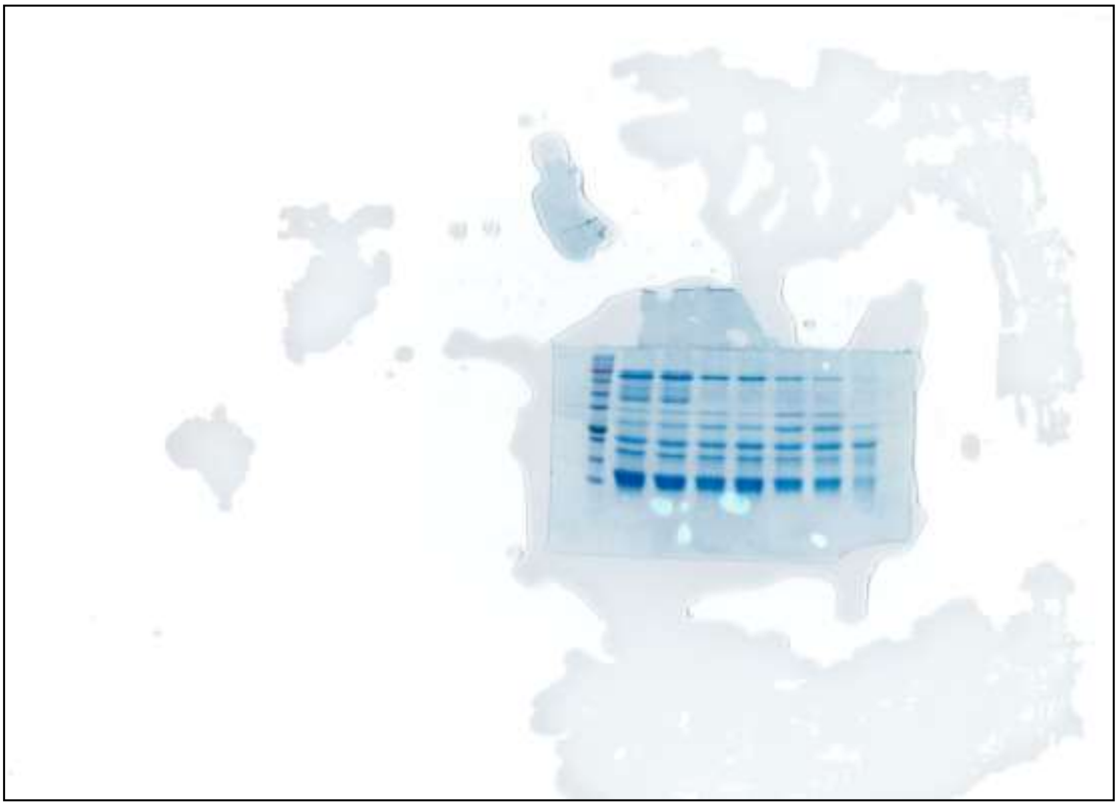

Link for Gel Analyzer 23.1 software

http://www.gelanalyzer.com/

Statistical analyses [SAS, 2004 (SAS Institute, Inc., Cary, NC)] is purchased version
